# Label-Free DNA Biosensor Based on Reduced Graphene Oxide and Gold Nanoparticles

**DOI:** 10.3390/bios13080797

**Published:** 2023-08-08

**Authors:** Elena Alina Chiticaru, Celina Maria Damian, Luisa Pilan, Mariana Ioniță

**Affiliations:** 1 Faculty of Medical Engineering, University Politehnica of Bucharest, Gh Polizu 1-7, 011061 Bucharest, Romania; elena.chiticaru@upb.ro; 2 Advanced Polymer Materials Group, University Politehnica of Bucharest, Gh Polizu 1-7, 011061 Bucharest, Romania; celina.damian@upb.ro; 3 Department of Inorganic Chemistry, Physical Chemistry and Electrochemistry, University Politehnica of Bucharest, Gh Polizu 1-7, 011061 Bucharest, Romania

**Keywords:** biosensor, electrochemistry, graphene, gold nanoparticles, DNA detection

## Abstract

Currently available DNA detection techniques frequently require compromises between simplicity, speed, accuracy, and cost. Here, we propose a simple, label-free, and cost-effective DNA detection platform developed at screen-printed carbon electrodes (SPCEs) modified with reduced graphene oxide (RGO) and gold nanoparticles (AuNPs). The preparation of the detection platform involved a two-step electrochemical procedure based on GO reduction onto SPCEs followed by the electrochemical reduction of HAuCl_4_ to facilitate the post-grafting reaction with AuNPs. The final sensor was fabricated by the simple physical adsorption of a single-stranded DNA (ssDNA) probe onto a AuNPs–RGO/SPCE electrode. Each preparation step was confirmed by morphological and structural characterization using scanning electron microscopy (SEM) and X-ray photoelectron spectroscopy, respectively. Furthermore, the electrochemical properties of the modified electrodes have been investigated by cyclic voltammetry (CV) and electrochemical impedance spectroscopy (EIS). The results demonstrated that the introduction of AuNPs onto RGO/SPCEs led to an enhancement in surface conductivity, a characteristic that favored an increased sensitivity in detection. The detection process relied on the change in the electrochemical signal induced by the binding of target DNA to the bioreceptor and was particularly monitored by the change in the charge transfer resistance of a [Fe(CN)_6_]^4–/3–^ redox couple added in the test solution.

## 1. Introduction

Point-of-care applications are moving toward more decentralized clinical diagnostic methods and approaches that are portable, selective, sensitive, highly effective, and produce accurate analytical results in a timely manner when compared to traditional laboratory testing; hence, they are attracting much attention in the biomedical field [1,2,3]. Over the past several years, the number of point-of-care testing platforms has steadily increased, aiding in their application in emergency and accident cases. There are many ways to identify illnesses and keep track of a patient’s health; however, biosensors have proven to play a crucial part in prognostics and diagnostics [4,5].

Particularly, electrochemical biosensors for DNA detection are analytical devices that use electrochemical principles to detect the presence and quantity of DNA molecules in a sample. These biosensors have emerged as a powerful tool for DNA analysis due to their high sensitivity, specificity, and rapid response time [6]. These devices have potential applications in a wide range of fields, including medical diagnosis, environmental monitoring, food safety, and forensic analysis [7,8,9,10]. In the biomedical field, synthetic DNA like peptide nucleic acids (PNA) has been used successfully in combination with graphenic species to fabricate sensitive electrochemical biosensors for the early detection of various diseases such as tuberculosis [11] or Brugada Syndrome [12]. In contrast, in the environmental monitoring field, it was shown that PNA biosensors could help manage pests by detecting specific gene expressions in plant bugs [13]. As research in this field continues, the development of new and improved electrochemical biosensors for DNA detection is likely to accelerate, providing new opportunities for DNA analysis and other biomedical applications.

Graphene and graphene-based nanomaterials have been showing the greatest promise among the nanomaterials applied in biosensors fabrication due to their outstanding properties like high surface area, excellent conductivity, chemical stability [14,15,16,17], and high biocompatibility with a wide range of biomolecules, including antibodies, enzymes, DNA, cells, and proteins [18,19,20,21]. Biosensors based on graphene have been applied in the detection of multiple analytes, from small molecules to biomolecules and even cells [22,23]. In particular, graphene-based biosensors have been extensively studied and optimized for selective detection and analysis of different DNA forms, enabling diverse applications in genomics, diagnostics, and DNA-based research [24,25]. Besides graphene, other nanomaterials have been employed for DNA detection to increase assay sensitivity. Gold nanostructures are excellent options among these nanomaterials because of their outstanding optical and mechanical characteristics, as well as their high chemical stability [26]. The synthesis of Au nanostructures can be achieved in a number of ways. Among these, the electrochemical synthesis of Au nanostructures has been widely employed because it offers several advantages over chemical synthesis, including the ability to control the size, shape, morphology, and growth rate by modulating the applied potential or current and via the electrolyte concentration [27,28,29]. Several studies have demonstrated that single-stranded (ss) oligonucleotides possess a more pronounced affinity for gold nanoparticles than their corresponding double-stranded (ds) helix [30,31]. Li and Rothberg mentioned that the underlying adsorption mechanism of ssDNA on gold is electrostatic and exploited the difference in the electrostatic properties of ssDNA and dsDNA in DNA detection by a colorimetric assay [30].

In this work, we aim to develop an electrochemical detection platform for DNA hybridization based on screen-printed carbon electrodes (SPCEs) modified with reduced graphene oxide (RGO) and gold nanoparticles (AuNPs). A protocol previously established by our group was employed for the preparation of RGO/SPCEs modified electrodes [32], and then, the functionalization of RGO with AuNPs was performed by the electrochemical reduction of chloroauric acid. The modified electrodes were extensively characterized before further modifications. Finally, the detection platform was obtained by the immobilization of the ssDNA probe (DNAp) at the AuNPs-RGO/SPCEs by simple physical adsorption, and the hybridization event between the ssDNA probe and ssDNA target (DNAt) was detected electrochemically. Cyclic voltammetry (CV), electrochemical impedance spectroscopy (EIS), and chronocoulometry were the electrochemical characterization and detection techniques, while the morphology of the films deposited at electrodes was determined by scanning electron microscopy (SEM). The results show successful detection of the DNA target for concentrations as low as 1 nM, while the specificity of the biosensor is demonstrated by control experiments using a non-complementary oligonucleotide (ncDNA).

The addition of AuNPs in the RGO/SPCE system improved the electrochemical properties of the platform, leading to a more sensitive detection, i.e., 100 times lower, compared with our previously obtained RGO/SPCE biosensor [32]. Similar biosensors were proposed for DNA detection [33,34,35], and a higher sensitivity was reported. However, these platforms are very complex, introducing in the fabrication protocol other materials like polypyrrole, L-cysteine, and intercalating electrochemical labels. Additionally, our electrochemical detection platform is easy to fabricate, does not require labels, is affordable, and it can be designed into a portable, miniaturized biosensor, compared to other systems that involve complex and time-consuming preparation steps [36], expensive equipment and reagents [37], or can analyze the sample only in controlled settings by professional personnel [38]. On the other hand, the sensitivity of our platform is lower compared to other similar but more intricate biosensors that have the capacity to detect low concentrations of analytes in complex biological samples [39]. Nonetheless, different groups reported other detection platforms, such as pencil graphite electrodes modified with polypyrrole, La_2_O_3_ nanoparticles, and snowflake-like Cu_2_S, with a similar or lower sensitivity than 1 nM [40]. In the current work, we aim to provide a simple, sensitive, and cost-efficient alternative to designing DNA biosensors.

## 2. Materials and Methods

### 2.1. Reagents and Materials

Graphene oxide (GO) dispersion in water (2 mg/mL), KCl, HCl, H_2_NaO_4_P, HNa_2_O_4_P, H_2_SO_4,_ and HAuCl_4_ were purchased from Sigma-Aldrich (St. Louis, MO, USA). Potassium ferricyanide (K_3_[Fe(CN)_6_]) and potassium ferrocyanide (K_4_[Fe(CN)_6_] × 3H_2_O) were obtained from Merck Co. (Darmstadt, Germany). All oligonucleotides, like ssDNA probe (5′-TTTCAACATCAGTCTGATAAGCTATCTCCC-3′), complementary ssDNA target (5′-GGGAGATAGCTTATCAGACTGATGTTGAAA-3′), along with Tris (10 mM)—EDTA (0.1 mM) called IDTE buffer were supplied from Integrated DNA Technologies, Inc. (Coralville, IA, USA). Screen-printed carbon electrodes (SPCE-DRP 110) were acquired from Metrohm DropSens, Spain, having integrated a carbon working electrode (WE) with a 4 mm diameter, a pseudo-reference electrode (silver), and a counter electrode (carbon). The Adrona Crystal EX water purification system was used for ultrapure water to wash the SPCEs after each modification step without compromising the integrity of the DNA molecules.

### 2.2. Procedures

#### 2.2.1. Morphological Characterization

The electrodes modified with AuNPs-RGO were characterized using a focused ion beam scanning electron microscope (FIB-SEM) and Versa 3D DualBeam instrument (FEI Company, Hillsboro, OR, USA) coupled with a TEAM EDS Analysis System (EDAX Inc., Pleasanton, CA, USA). The secondary electrons signals were detected in a high-vacuum functioning mode (6.1 × 10^–4^ Pa) at a 10 mm distance, an accelerating voltage of 10 kV, and a 4.5 spot size, which allowed the investigation of the plane view (0° tilt) electrodes surface morphology. The image stability was ensured using SmartSCAN software and the DCFI drift suppression options of the Versa 3D DualBeam instrument.

#### 2.2.2. Electrochemical Characterization

Using the potentiostat Autolab PGSTAT 204 model (Metrohm Autolab, Utrecht, The Netherlands) and the NOVA 2.1 software (Metrohm Autolab), each stage of the electrode modification process was examined by CV and EIS at room temperature. The SPCE was coupled to a connector (DSC) purchased from Metrohm Dropsens, operating as a link between the potentiostat and SPCEs. Unless stated otherwise, the electrochemical evaluations were performed in a 100 µL 0.1 M KCl electrolyte solution and 1 mM K_3_[Fe(CN)_6_]/K_4_[Fe(CN)_6_] (1:1) redox system. CV curves were obtained in the potential range of –0.2 V and +0.6 V at a sweep rate of 0.05 V/s. The impedimetric measurements were recorded at the formal potential of the redox probes, with a 10 mV alternating current (AC) amplitude, in the frequency window of 0.01–10^5^ Hz.

#### 2.2.3. Electrode Functionalization Procedure

The SPCEs were cleaned with ultrapure water prior to any modification. Then, they received an electrochemical pretreatment to enhance their electrochemical properties and to improve the hydrophilicity of the carbon WE. To this end, five CV cycles were performed first in 0.1 M HCl, applying a potential range of +0.5 V and −1.5 V at a 0.05 V/s scan rate, followed by five voltammetric cycles in a 0.1 M phosphate buffer solution (PBS), pH 7, between 0 and +2 V, at the same scan rate. Next, the electrodes were covered with 3 µL PBS, dried in an oven at 60 °C for 10 min, and washed with ultrapure water.

Following the cleaning and drying of the electrodes, a 3 µL GO dispersion (0.3 mg/mL) was deposited on the WE with an electronic micropipette. GO-modified electrodes (GO/SPCE) were then left at 60 °C for two hours and at room temperature until the next day. Moreover, 100 µL of 0.5 M KCl was added on the surface of GO/SPCEs, and ten CV cycles were applied from 0 to −1.5 V, with a sweep rate of 0.1 V/s to reduce GO electrochemically.

The functionalization procedure of RGO/SPCEs with AuNPs consisted in performing five CV cycles in a potential range from +1 to −1 V, at a sweep rate of 0.05 V/s, in a solution containing various concentrations (1, 5, 10, or 15 mM) of HAuCl_4_ in 0.5 mM H_2_SO_4_. The SPCEs were modified at the same time, and three samples were used for each test in order to attest to the reproducibility and validity of the results.

#### 2.2.4. The Fabrication and Testing Procedure of the DNA Biosensor

The bioreceptor consisting of an ssDNA probe was immobilized at AuNPs-RGO/SPCEs through non-covalent bonding (physical adsorption) by incubating the electrodes with 10 µL of a 500 nM DNA solution overnight at room temperature. The ssDNA/AuNPs-RGO/SPCEs biosensor, fabricated as previously described, was then tested by measuring the electrochemical changes that occurred after the hybridization with a complementary ssDNA target. The hybridization was performed by incubating the sensor with a DNA target solution for 3 h at 42 °C. The specificity of the biosensor was tested by incubating it with non-complementary DNA in the exact same conditions used for the hybridization with the DNA target (3 h at 42 °C).

#### 2.2.5. Chronocoulometric Tests

Chronocoulometry has been used to estimate the DNA surface coverage before and after the target DNA addition as indicative of the hybridization process by measuring the reduction charge of the accumulated cationic Ru(NH_3_)_6_^3+^ redox species with the negatively charged backbone of the surface-bound oligonucleotides. The applied two-step chronocoulometric procedure consisted in stepping from 0 to −0.400 V to 0 V vs. Ag/AgCl, with a pulse period of 0.250 s. In this regard, the electrode is immersed in a deaerated low-ionic strength buffer solution (TRIS 10 mM, pH = 7.4) and maintained for 10 min under stirring, for equilibration, before performing the chronocoulometric test. The same procedure is then applied by the addition of 100 μM Ru(NH_3_)_6_^3+^ to the buffer solution. The DNA surface coverages were calculated using a previously established method based on the integrated Cottrell expression [41]. Thus, the redox marker surface excess (Γ_0_) is determined from the difference in chronocoulometric intercepts in the representation of the total charge (Q) as a function of the square root of time (t^1/2^) for the identical experiment in the presence and absence of redox marker: Γ_0_ = ∆Q/nFA, with *n* = 1, the number of electrons transferred, F = 96,485 C mol^−1^, Faraday constant, and A the electrode area in cm^2^. Then, Γ_DNA_, the probe surface density in molecules/cm^2^, can be calculated as Γ_DNA_ = Γ_0_ z/m, with m = the number of bases in the probe DNA and z the charge of the redox molecule.

## 3. Results and Discussion

### 3.1. Morphological Characterization

**Figure 1:**
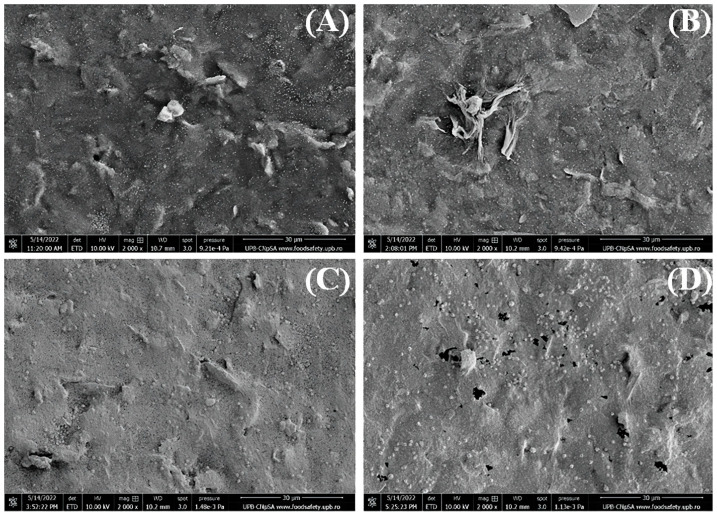
SEM images recorded at 2000× magnification (30 µm scale) for RGO/SPCEs functionalized with AuNPs from (**A**) 1 mM, (**B**) 5 mM, (**C**) 10 mM, and (**D**) 15 mM HAuCl_4_ solutions in 0.5 mM H_2_SO_4_.

The morphology of the electrodes modified with graphene and different concentrations of gold nanoparticles was investigated by SEM to determine the influence of increasing AuNPs concentration. Several images were recorded at different magnifications, and the most representative ones are shown below. First, the images are presented at 2000× magnification (Figure 1) to observe the coverage of RGO/SPCE with gold nanoparticles. It is easily noticeable that the modified electrode surface is covered with a moderate quantity of 1 mM AuNPs (Figure 1A), and the RGO sheet can still be observed. When 5 mM HAuCl_4_ was used for SPCE modification, more nanoparticles formed on the graphene electrode and were uniformly dispersed on the entire surface (Figure 1B), while increasing the concentration to 10 mM determined the whole electrode surface to be covered with AuNPs that also show a small tendency for agglomeration (Figure 1C). Finally, at 15 mM HAuCl_4_, the RGO surface is also entirely covered with AuNPs, while small dark patches can also be observed that seem to be material stripped off the carbon substrate (Figure 1D).

**Figure 2:**
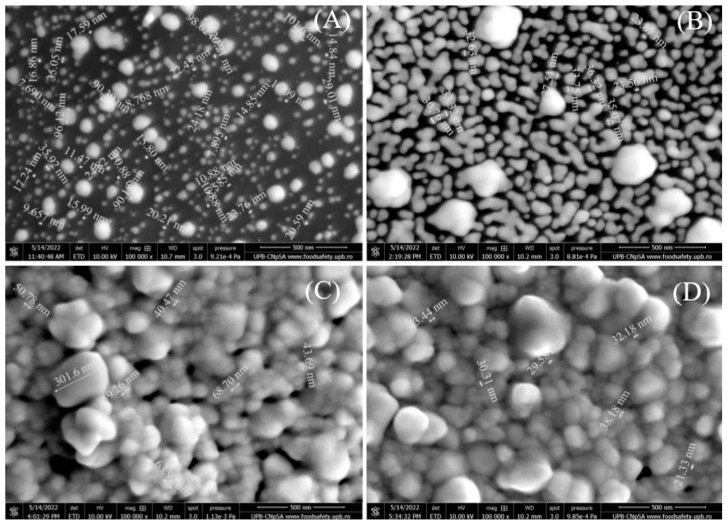
SEM images recorded at 100,000× magnification (500 nm scale) for RGO/SPCEs functionalized with AuNPs from (**A**) 1 mM, (**B**) 5 mM, (**C**) 10 mM, and (**D**) 15 mM HAuCl_4_ solutions in 0.5 mM H_2_SO_4_.

Secondly, the SEM images are also presented at 100,000× magnification (Figure 2) to better observe the morphology and size of the gold nanoparticles. Figure 2A shows the SPCE functionalized with 1 mM HAuCl_4,_ and it is revealed that the RGO substrate is covered with AuNPs between 7 and 90 nm in size. Increasing the concentration to 5 mM (Figure 2B), the number of nanoparticles increased, covering more of the electrode surface and showing nanoparticles of 23–33 nm, but also a few agglomerated ones. At 10 mM (Figure 2C) and 15 mM (Figure 2D), the SEM images are very similar, showing that AuNPs covered the entire SPCE surface and that the nanoparticles increase in size (up to 300 nm) and have a higher tendency of agglomeration.

### 3.2. Structural Characterization

**Figure 3:**
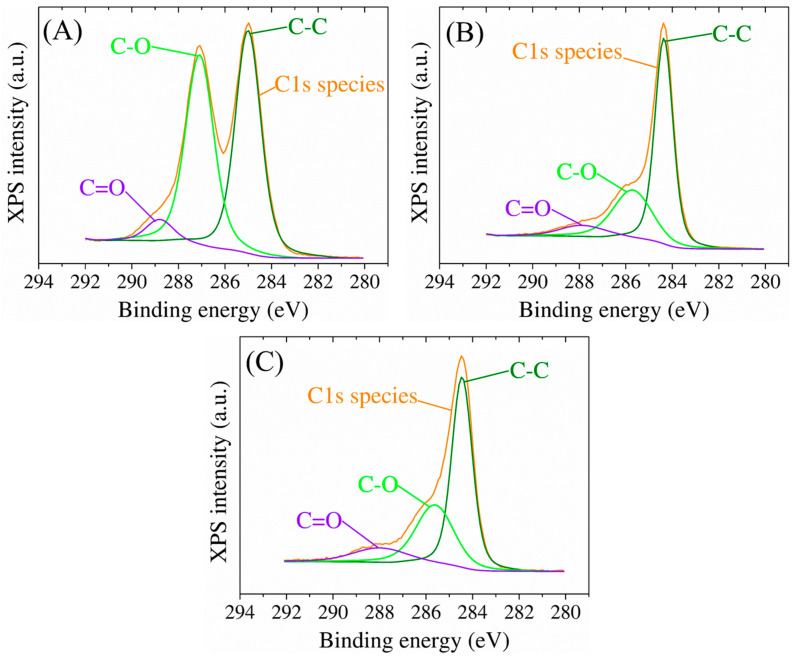
High-resolution C1 XPS spectra of bare SPCE modified with (**A**) GO, (**B**) RGO, and (**C**) AuNPs. The orange curve represents the C1s high-resolution spectrum of GO, RGO, and AuNPs-RGO thin layer, respectively, on the surface of the electrode.

**Figure 4:**
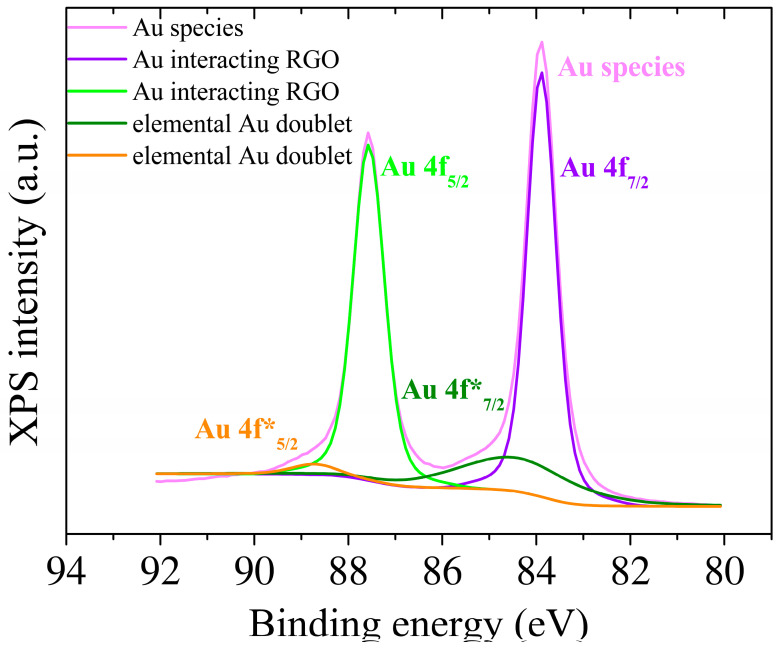
High-resolution XPS Au 4f spectra of SPCE modified with RGO and AuNPS.

XPS was used to conduct a comprehensive analysis of the elemental composition of the surface of the modified electrodes (Figure 3). The survey spectra of GO-modified SPCE indicated the presence of carbon and oxygen species, with small amounts of nitrogen detected due to process contamination. The C to O atomic ratio from the XPS survey spectra was used as a reference to assess the presence of oxygenated functional groups in the GO sheets. The assessment revealed that the GO samples had a high percentage of oxygen atoms, as indicated by a C:O ratio value of 2.7. Upon electrochemical reduction, the signals for oxygen-containing functional groups decreased significantly, indicating an efficient reduction of GO to RGO. The C:O atomic ratio increased to 6.2 in the RGO samples, indicating that the delocalized π conjugation was partially restored, as the relatively low intensity of the C-O peak from 286 eV (Figure 3A,B) suggests, compared to that obtained from the GO surfaces, which is also consistent with the C:O ratio, as it was observed through deconvolution of the experimental C1s high-resolution data (orange curve), confirming the occurrence of different functional groups in the RGO thin film. Moreover, the addition of gold nanoparticles is confirmed by this characterization technique which shows a content of approximately 10.4% Au on the surface of the electrode and a decrease of the C:O ratio to 5.3, offering insight into the C:O:Au atomic distribution upon the modified electrode surface. In the case of the presence of gold nanoparticles, only a slight shift towards lower binding energies is observed due to an electropositive electron interaction between RGO and Au as it is translated in the C-C secondary peak position (Figure 3C). According to Moulder [42], the secondary peaks for Au nanoparticles can be assigned to elemental Au 4f energy levels as well as a small part of these Au species were noticed to interact with the aromatic structure of RGO, translated as the Au* 4f shifted bands (Figure 4).

### 3.3. Electrochemical Characterization

#### 3.3.1. AuNPs-RGO Functionalized SPCEs

**Figure 5:**
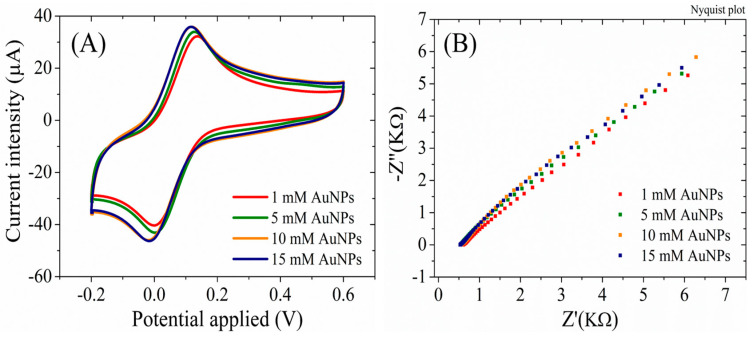
(**A**) CV and (**B**) EIS Nyquist plot recorded in 1 mM [Fe(CN)_6_]^3–/4–^, 0.1M KCl, for RGO/SPCE functionalized with AuNPs from 1 mM, 5 mM, 10 mM, and 15 mM HAuCl_4_ solutions in 0.5 mM H_2_SO_4_.

The RGO/SPCEs functionalized with AuNPs, fabricated from HAuCl_4_ solutions of different concentrations, have also been investigated electrochemically using cyclic voltammetry (Figure 5A) and electrochemical impedance spectroscopy techniques in the presence of 1 mM [Fe(CN)_6_]^3–/4–^ redox species in a 0.1 M KCl solution (Figure 5B). The CV studies indicate a slight increase in the characteristic peak current intensity for an increase of the HAuCl_4_ concentration in the synthesis solution up to 10 mM. As displayed in Figure 5A, for electrodes fabricated with 10 and 15 mM HAuCl_4_, the CVs overlap confirming the SEM results that indicated a surface completely covered with AuNPs at 10 mM and no further change in the surface properties for higher concentrations. Similarly, the EIS measurements support these results, displaying an absence of the semicircle in the Nyquist plot, indicative of a very low charge transfer resistance (Rct) for all AuNPs-RGO/SPCEs.

Considering the morphological (Figure 1 and Figure 2) and electrochemical (Figure 5) characterizations that showed complete coverage of the RGO/SPCE with AuNPs at 10 mM and no significant changes at 15 mM, the biosensor was developed using 10 mM HAuCl_4_ to graft AuNPs on the RGO/SPCE surface.

**Figure 6:**
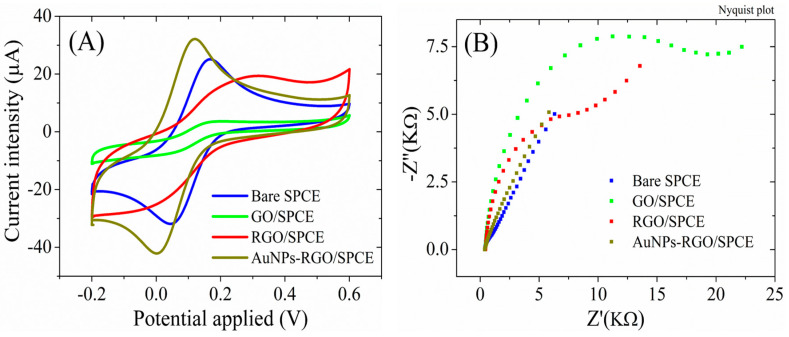
(**A**) CV and (**B**) EIS Nyquist plot recorded in 1 mM [Fe(CN)_6_]^3–/4–^, 0.1 M KCl, for bare SPCE, GO/SPCE, RGO/SPCE, and AuNPs-RGO/SPCE.

The electrochemical properties were also recorded for each stage of SPCE functionalization. In Figure 6, we present the typical voltammograms recorded for the tested samples after each modification step. The CV measurements for the bare SPCE in the presence of 1 mM [Fe(CN_6_)]^3–/4–^ redox species (Figure 6A) display well-defined redox peaks with a current intensity of 26.05 ± 0.39 µA and peak-to-peak separation (ΔEp) below 100 mV, indicating a good electrical conductivity for this substrate. Deposition of the low-conductive GO dispersion induced a substantial change in the CVs, with the intensity of the peak currents decreasing down to 3.75 ± 0.16 µA. After the electrochemical reduction of GO, the peak current intensity increases up to 21.11 ± 0.61 µA, while the CVs displayed higher capacitive currents, as expected for electrodes with an increased electrochemically active surface area. Finally, the modification of the RGO surface with gold nanoparticles induced a significant change in the CV shape, with more defined peaks, increased redox signals (reaching 32.61 ± 0.97 µA), and a lower ΔEp. These characteristics suggest an improved conductivity of the AuNPs-RGO/SPCE-modified electrode.

The impedimetric results (Figure 6B) are in accordance with the CV measurements. While the Nyquist spectrum for the bare SPCE is defined by the presence of a Warburg line and a low charge transfer resistance, the electrode modification with GO caused the formation of a large semicircle indicative of a high Rct (15.2 ± 1.8 KΩ) that decreased after the GO electrochemical reduction to 7.7 ± 0.7 KΩ, as a result of the better conductivity of RGO compared to GO. The complete disappearance of the semicircle and the presence of the simple diffusion line for AuNPs-RGO/SPCE confirms the superior conductivity of the complex graphene–gold nanoparticles.

**Figure 7:**
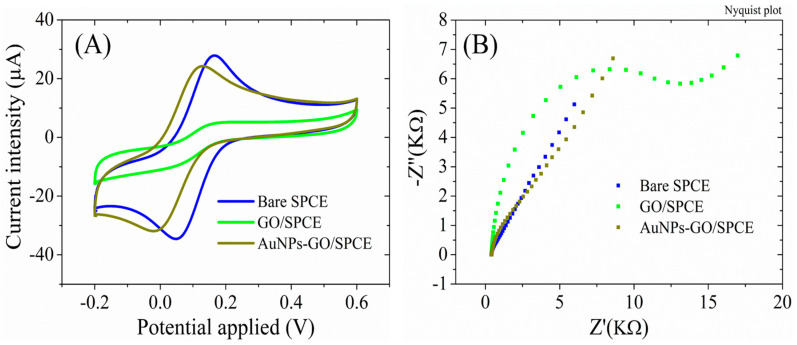
(**A**) CV and (**B**) EIS Nyquist plot recorded in 1 mM [Fe(CN)_6_]^3–/4–^, 0.1 M KCl, for bare SPCE, GO/SPCE, and AuNPs-GO/SPCE.

The necessity of GO electrochemical reduction before AuNPs functionalization is revealed by the electrochemical properties of the SPCEs modified with GO dispersion and AuNPs in comparison with AuNPs-RGO/SPCEs. Accordingly, the AuNPs-GO/SPCE system displayed lower peak currents (Figure 7A) of only 25 ± 0.98 µA, compared to the 32.61 ± 0.97 µA previously obtained for AuNPs-RGO/SPCEs. Moreover, a higher impedance was observed for the AuNPs-GO/SPCE system in EIS measurements (Figure 7B), despite the same increase in Rct after modification of bare SPCEs with GO and the disappearance of the semicircle in the Nyquist plot after the modification with AuNPs (indicating the conductivity improvement).

**Figure 8:**
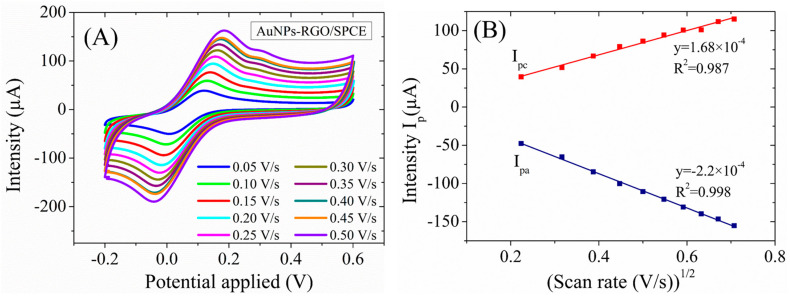
(**A**) CV curves of AuNPs-RGO/SPCE at different scan rates in 1 mM [Fe(CN)_6_]^3–/4–^ solution with 0.1M KCl. (**B**) Plot of anodic and cathodic peaks current vs. square root of scan rates.

A study of the kinetics of the [Fe(CN)_6_]^3–/4–^ redox probe at the fabricated AuNPs-RGO/SPCE electrodes was also performed. To this end, CV has been performed at various scan rates, ranging from 0.05 to 0.5 V/s (Figure 8A), and the expected linear relationship with the square root of the scan rate is observed (Figure 8B). This implies a diffusion-controlled oxido-reduction process at the electrode surface, in agreement with the Randles–Ševčík equation for quasi-reversible one-electron transfer processes. The use of the Randles–Ševčík equation to accurately determine the real surface area for the electrodes modified with nanomaterials from CV experiments is required to be considered with care [43,44]; therefore, we emphasize that the surface area of AuNPs-GO/SPCE-modified electrode determined here to be on average 0.21 cm^2^, based on this equation, is only an estimation.

#### 3.3.2. The Characterization and Testing of the DNAp/AuNPs-RGO/SPCE Sensing Platform

**Figure 9:**
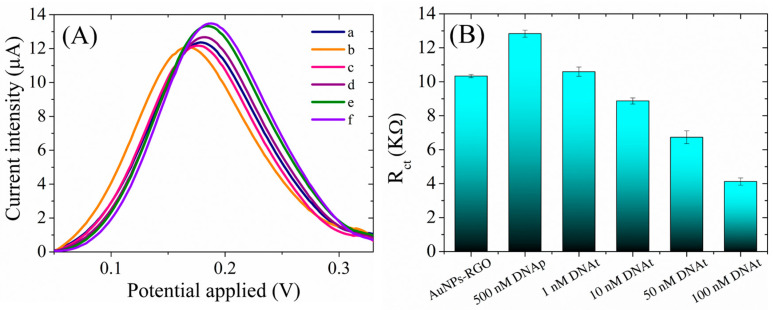
(**A**) Anodic peaks in CV (after baseline correction) recorded in 1 mM [Fe(CN)_6_]^3–/4–^, 0.1 M KCl, for (a) AuNPs-RGO/SPCE modified with (b) 500 nM DNA probe and hybridized with (c) 1 nM, (d) 10 nM, (e) 50, and (f) 100 nM DNA target. (**B**) Charge transfer resistance recorded by EIS in 1 mM [Fe(CN)_6_]^3–/4–^, 0.1 M KCl, for AuNPs-RGO/SPCE modified with 500 nM DNA probe and hybridized with 1 nM, 10 nM, 50, and 100 nM DNA target.

The preparation of the biosensor by the immobilization of the ssDNA probe, as a bioreceptor, at the detection interface consisted in the incubation of the AuNPs-RGO/SPCEs with 500 nM ssDNA probe at room temperature overnight. The hybridization tests, in the presence of the complementary DNA target, were performed by incubating the newly prepared DNAp/AuNPs-RGO/SPCE sensor with different concentrations of a DNA target (1 nM–100 nM) at 42 °C for 3 h. Figure 9 displays the change in the electrochemical properties of the interface after each step in the preparation and testing of the final sensor, tested in a solution containing 1 mM [Fe(CN)_6_]^3–/4–^ redox species in 0.1M KCl. Accordingly, a first decrease in the oxidation peak current was observed after DNA probe immobilization, which is to be expected because the oligonucleotide is a large molecule that blocks the electron transfer between the electrolyte solution and electrode surface (Figure 9A). Next, the incubations of the sensor with 1 nM, 10 nM, 50 nM, and 100 nM DNA targets determined successive increases in current intensity, which can be explained by the formation of dsDNA with each addition of a DNA target. In agreement with the previous findings [31], we may assume that the newly formed dsDNA easily detaches from the surface, thus favoring the redox process of [Fe(CN)_6_]^3–/4–^ species. The impedimetric measurements (Figure 9B) were in agreement with the CV results and showed an increase in Rct following the adsorption of the DNA probe at the AuNPs-RGO/SPCE interface, which impeded the redox process of the negatively charged [Fe(CN)_6_]^3–/4–^. Moreover, as expected from the CV results, the hybridization with the DNA target determined a decrease in Rct for each successive addition up to 100 nM. Both the increase in the anodic oxidation current and the decrease in the charge transfer resistance imply changes in the heterogeneous electron transfer of [Fe(CN)_6_]^3–/4–^ at the sensing interface as a result of the hybridization process that can be exploited in DNA detection.

The specificity of the biosensor was also demonstrated by control experiments using a non-complementary oligonucleotide. Thus, the incubation of DNAp-AuNPs-RGO/SPCE with non-complementary DNA induced negligible change in the electrochemical signal, both in CV (0.3 μA increase in current intensity for ncDNA, in comparison with 4.4 μA for DNAt) and EIS (0.32 kΩ for ncDNA, in comparison with 8.26 kΩ for DNAt) for a 100 nM concentration of DNA. The subsequent addition, in the same experiment, of the complementary DNA target determined a predictable increase in the current intensity in CV, and Rct decrease in EIS, suggesting that the detection platform can discriminate between specific DNA sequences.

#### 3.3.3. Chronoculometry Measurements

**Figure 10:**
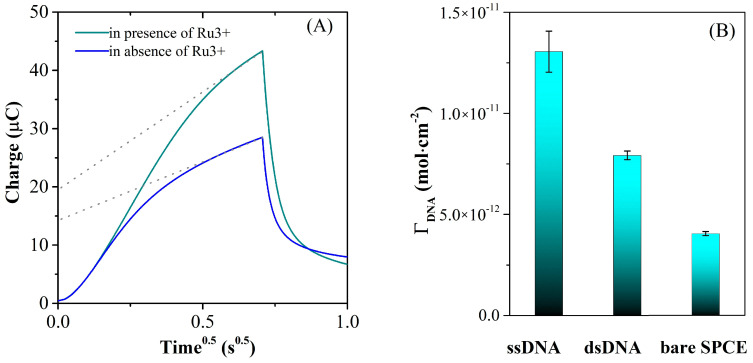
(**A**) Typical chronocoulometric curves illustrated for dsDNA/AuNPs-RGO/SPCE electrodes using 100 μM Ru(NH_3_)^3+^ as a redox indicator. (**B**) The calculated oligonucleotides surface density before (ssDNA) and after hybridization with 100 nM DNAt (dsDNA) suggests the formation of dsDNA after DNA target addition, with lower affinity for the modified SPCE surface.

The surface density of DNA-modified electrodes can be quantified using chronocoulometry by taking advantage of the electrostatic attraction between specific redox cations added in the test solution and the nucleotide phosphate backbone of DNA. Redox molecules can interact with DNA through either electrostatic attraction or intercalation, and the nature of the interaction is often influenced by the ionic strength and molecular structure of the redox molecule [41]. Therefore, in order to perform the measurement, the DNA-modified electrodes were placed in a low-ionic-strength electrolyte solution (TRIS 10 mM, pH = 7.4) containing 100 μM Ru(NH_3_)_6_^3+^ cationic redox marker. The redox cations in the solution replace the native counterions that are associated with the nucleotide phosphate residues of the DNA probe, and the amount of redox marker that attaches electrostatically to the DNA-modified electrode is then determined using chronocoulometry. When the redox marker reaches saturation coverage, the surface density of the DNA probe is calculated by assuming that the DNA phosphate residues are completely compensated by the redox cations. This approach is advantageous because it is insensitive to variations in base composition and chain order (single-stranded versus duplex), unlike other non-covalent labeling techniques [45]. The system is observed under equilibrium conditions so that the electrodes have been immersed in the deaerated buffer solution and maintained for 10 min under stirring, for equilibration, before performing the chronocoulometric test. The DNA surface coverages were calculated using the established method reported by Steel et al. [41]. Thus, Figure 10A presents the typical chronocoulometric response that affords the calculation of the surface concentration of the oligonucleotide present at the electrode from the difference in chronocoulometric intercepts (nFAΓ_0_), assuming that for the measurements performed both in the absence and presence of Ru(NH_3_)_6_^3+^ the double layer capacitance has the same value. Considering a complete charge compensation of DNA by redox marker, the conversion of the calculated Γ_0_ to DNA surface density (Γ_DNA_) was made as indicated in the experimental part, and the Γ_DNA_ values before (ssDNA) and after hybridization with 100 nM DNAt (dsDNA) are presented in Figure 10B. The results correlate well with the CV and EIS data presented previously and demonstrate the removal from the electrode surface of the hybridized dsDNA after DNA target addition.

## 4. Conclusions

A simple, cost-effective biosensor employing the AuNPs-RGO nanocomposite as a transducing interface was developed for the direct detection of DNA hybridization. The electrochemical platform has good sensitivity, can detect DNA molecules as low as 1 nM, and can discriminate between complementary and non-complementary DNA. Based on DNA complementarity and our experimental results, one can say that the proposed biosensor has the potential to demonstrate a high selectivity in biological samples. Additional studies are intended to demonstrate the high selectivity of the biosensor in complex biological samples. The detection of DNA hybridization was demonstrated by several electrochemical techniques, such as CV, EIS, and chronocoulometry. The sensitivity of 1 nM for the studied biosensor could be suitable for certain medical purposes, depending on the specific application and the concentration of the target biomarker or analyte being detected. However, this is a proof-of-concept study, and further thorough investigations will be made in order to improve the sensitivity and reproducibility of the biosensor. The results obtained in this work can pave the way towards the fabrication of DNA biosensors at an industrial scale with applicability in various medical applications due to the high biocompatibility and stability of graphene functionalized with gold nanoparticles.

## Data Availability

Not applicable.

## References

[1] Roy L., Buragohain P., Borse V. (2022). Strategies for sensitivity enhancement of point-of-care devices. Biosens. Bioelectron. X.

[2] Stranieri A., Venkatraman S., Minicz J., Zarnegar A., Firmin S., Balasubramanian V., Jelinek H.F. (2022). Emerging point of care devices and artificial intelligence: Prospects and challenges for public health. Smart Health.

[3] Lopes L.C., Santos A., Bueno P.R. (2022). An outlook on electrochemical approaches for molecular diagnostics assays and discussions on the limitations of miniaturized technologies for point-of-care devices. Sens. Actuators Rep..

[4] Lino C., Barrias S., Chaves R., Adega F., Martins-Lopes P., Fernandes J.R. (2022). Biosensors as diagnostic tools in clinical applications. Biochim. Et Biophys. Acta (BBA)-Rev. Cancer.

[5] Mahshid S.S. Electrochemical Immuno-Biosensors on Nanostructured Electrodes for Rapid Sensitive Detection of Disease Biomarkers. Proceedings of the Electrochemical Society Meeting Abstracts 239.

[6] Zhang L., Su W., Liu S., Huang C., Ghalandari B., Divsalar A., Ding X. (2022). Recent Progresses in Electrochemical DNA Biosensors for MicroRNA Detection. Phenomics.

[7] Li T., Shang D., Gao S., Wang B., Kong H., Yang G., Shu W., Xu P., Wei G. (2022). Two-Dimensional Material-Based Electrochemical Sensors/Biosensors for Food Safety and Biomolecular Detection. Biosensors.

[8] Hosseini S.S., Jebelli A., Vandghanooni S., Jahanban-Esfahlan A., Baradaran B., Amini M., Bidar N., de la Guardia M., Mokhtarzadeh A., Eskandani M. (2022). Perspectives and trends in advanced DNA biosensors for the recognition of single nucleotide polymorphisms. J. Chem. Eng..

[9] Zahra Q.u.A., Fang X., Luo Z., Ullah S., Fatima S., Batool S., Qiu B., Shahzad F. (2022). Graphene Based Nanohybrid Aptasensors in Environmental Monitoring: Concepts, Design and Future Outlook. Crit. Rev. Anal. Chem..

[10] Bilge S., Dogan-Topal B., Gürbüz M.M., Yücel A., Sınağ A., Ozkan S.A. (2022). Recent advances in electrochemical sensing of cocaine: A review. TrAC Trends Anal. Chem..

[11] Mat Zaid M.H., Abdullah J., Yusof N.A., Sulaiman Y., Wasoh H., Md Noh M.F., Issa R. (2017). PNA biosensor based on reduced graphene oxide/water soluble quantum dots for the detection of Mycobacterium tuberculosis. Sens. Actuators B Chem..

[12] Moretta R., Terracciano M., Borbone N., Oliviero G., Schiattarella C., Piccialli G., Falanga A.P., Marzano M., Dardano P., De Stefano L. (2020). PNA-Based Graphene Oxide/Porous Silicon Hybrid Biosensor: Towards a Label-Free Optical Assay for Brugada Syndrome. Nanomaterials.

[13] Tian W., Zhang T., Gu S., Guo Y., Gao X., Zhang Y. (2021). OBP14 (Odorant-Binding Protein) Sensing in *Adelphocoris lineolatus* Based on Peptide Nucleic Acid and Graphene Oxide. Insects.

[14] Amărandi R.-M., Becheru D.F., Vlăsceanu G.M., Ioniță M., Burns J.S. (2018). Advantages of Graphene Biosensors for Human Stem Cell Therapy Potency Assays. BioMed Res. Int..

[15] Morales-Narváez E., Baptista-Pires L., Zamora-Gálvez A., Merkoçi A. (2017). Graphene-Based Biosensors: Going Simple. Adv. Mater..

[16] Chauhan N., Maekawa T., Kumar D.N.S. (2017). Graphene based biosensors—Accelerating medical diagnostics to new-dimensions. J. Mater. Res..

[17] Janegitz B.C., Silva T.A., Wong A., Ribovski L., Vicentini F.C., Taboada Sotomayor M.d.P., Fatibello-Filho O. (2017). The application of graphene for in vitro and in vivo electrochemical biosensing. Biosens. Bioelectron..

[18] Kyriakides T.R., Raj A., Tseng T.H., Xiao H., Nguyen R., Mohammed F.S., Halder S., Xu M., Wu M.J., Bao S. (2021). Biocompatibility of nanomaterials and their immunological properties. Biomed. Mater..

[19] Kucherenko I.S., Soldatkin O.O., Kucherenko D.Y., Soldatkina O.V., Dzyadevych S.V. (2019). Advances in nanomaterial application in enzyme-based electrochemical biosensors: A review. Nanoscale Adv..

[20] Nicolson F., Ali A., Kircher M.F., Pal S. (2020). DNA Nanostructures and DNA-Functionalized Nanoparticles for Cancer Theranostics. Adv. Sci..

[21] Jamalipour Soufi G., Iravani S. (2020). Eco-friendly and sustainable synthesis of biocompatible nanomaterials for diagnostic imaging: Current challenges and future perspectives. Green Chem..

[22] Wang Y., Li Z., Wang J., Li J., Lin Y. (2011). Graphene and graphene oxide: Biofunctionalization and applications in biotechnology. Trends Biotechnol..

[23] Liu J., Tang J., Gooding J.J. (2012). Strategies for chemical modification of graphene and applications of chemically modified graphene. J. Mater. Chem..

[24] Park J.S., Goo N.-I., Kim D.-E. (2014). Mechanism of DNA Adsorption and Desorption on Graphene Oxide. Langmuir.

[25] Ferapontova E.E. (2018). DNA Electrochemistry and Electrochemical Sensors for Nucleic Acids. Annu. Rev. Anal. Chem..

[26] Ielo I., Rando G., Giacobello F., Sfameni S., Castellano A., Galletta M., Drommi D., Rosace G., Plutino M.R. (2021). Synthesis, Chemical–Physical Characterization, and Biomedical Applications of Functional Gold Nanoparticles: A Review. Molecules.

[27] Xiao T., Huang J., Wang D., Meng T., Yang X. (2020). Au and Au-Based nanomaterials: Synthesis and recent progress in electrochemical sensor applications. Talanta.

[28] Evers M.V., Bernal M., Roldan Cuenya B., Tschulik K. (2019). Piece by Piece—Electrochemical Synthesis of Individual Nanoparticles and their Performance in ORR Electrocatalysis. Angew. Chem. Int. Ed..

[29] Shamsabadi A.S., Tavanai H., Ranjbar M., Farnood A., Bazarganipour M. (2020). Electrochemical non-enzymatic sensing of glucose by gold nanoparticles incorporated graphene nanofibers. Mater. Today Commun..

[30] Li H., Rothberg L. (2004). Colorimetric detection of DNA sequences based on electrostatic interactions with unmodified gold nanoparticles. Proc. Natl. Acad. Sci. USA.

[31] Gorbunova E.A., Epanchintseva A.V., Pyshnyi D.V., Pyshnaya I.A. (2023). Noncovalent Adsorption of Single-Stranded and Double-Stranded DNA on the Surface of Gold Nanoparticles. Appl. Sci..

[32] Chiticaru E.A., Pilan L., Damian C.-M., Vasile E., Burns J.S., Ioniţă M. (2019). Influence of Graphene Oxide Concentration when Fabricating an Electrochemical Biosensor for DNA Detection. Biosensors.

[33] Fani M., Rezayi M., Pourianfar H.R., Meshkat Z., Makvandi M., Gholami M., Rezaee S.A. (2021). Rapid and label-free electrochemical DNA biosensor based on a facile one-step electrochemical synthesis of rGO–PPy–(L-Cys)–AuNPs nanocomposite for the HTLV-1 oligonucleotide detection. Biotechnol. Appl. Biochem..

[34] Fani M., Rezayi M., Meshkat Z., Rezaee S.A., Makvandi M., Angali K.A. (2020). A Novel Electrochemical DNA Biosensor Based on a Gold Nanoparticles-Reduced Graphene Oxide-Polypyrrole Nanocomposite to Detect Human T-Lymphotropic Virus-1. IEEE Sens. J..

[35] Safarzadeh M., Pan G. (2022). Detection of a Double-Stranded MGMT Gene Using Electrochemically Reduced Graphene Oxide (ErGO) Electrodes Decorated with AuNPs and Peptide Nucleic Acids (PNA). Biosensors.

[36] Chen J., Wang M., Zhou X., Nie Y., Su X. (2021). Highly sensitive label-free fluorescence determination of lymphotropic virus DNA based on exonuclease assisted target recycling amplification and in-situ generation of fluorescent copper nanoclusters. Sens. Actuators B Chem..

[37] Wu Y., Dang H., Park S.-G., Chen L., Choo J. (2022). SERS-PCR assays of SARS-CoV-2 target genes using Au nanoparticles-internalized Au nanodimple substrates. Biosens. Bioelectron..

[38] Chang D., Tram K., Li B., Feng Q., Shen Z., Lee C.H., Salena B.J., Li Y. (2017). Detection of DNA Amplicons of Polymerase Chain Reaction Using Litmus Test. Sci. Rep..

[39] Lin X., Lian X., Luo B., Huang X.-C. (2020). A highly sensitive and stable electrochemical HBV DNA biosensor based on ErGO-supported Cu-MOF. Inorg. Chem. Commun..

[40] Foroughi M.M., Jahani S. (2022). Investigation of a high-sensitive electrochemical DNA biosensor for determination of Idarubicin and studies of DNA-binding properties. Microchem. J..

[41] Steel A.B., Herne T.M., Tarlov M.J. (1998). Electrochemical Quantitation of DNA Immobilized on Gold. Anal. Chem..

[42] Moulder J.F., Stickle W.F., Sobol W.M., Bomben K.D. (1992). Handbook of X-ray Photoelectron Spectroscopy.

[43] Bogdanowicz R., Ficek M., Malinowska N., Gupta S., Meek R., Niedziałkowski P., Rycewicz M., Sawczak M., Ryl J., Ossowski T. (2020). Electrochemical performance of thin free-standing boron-doped diamond nanosheet electrodes. J. Electroanal. Chem..

[44] Paixão T.R.L.C. (2020). Measuring Electrochemical Surface Area of Nanomaterials versus the Randles−Ševčík Equation. ChemElectroChem.

[45] Piehler J., Brecht A., Gauglitz G., Zerlin M., Maul C., Thiericke R., Grabley S. (1997). Label-Free Monitoring of DNA–Ligand Interactions. Anal. Biochem..

